# Dynamic Input Deep Learning Control of Artificial Avatars in a Multi-Agent Joint Motor Task

**DOI:** 10.3389/frobt.2021.665301

**Published:** 2021-08-09

**Authors:** Maria Lombardi, Davide Liuzza, Mario di Bernardo

**Affiliations:** ^1^ Department of Engineering Mathematics, University of Bristol, Bristol, United Kingdom; ^2^ Department of Electrical Engineering and Information Technology, University of Naples Federico II, Naples, Italy; ^3^ ENEA Fusion and Nuclear Safety Department, Frascati, Italy; ^4^ Scuola Superiore Meridionale, University of Naples Federico II, Naples, Italy

**Keywords:** reinforcement learning, nonlinear control, human-robot interaction, virtual player, mirror game, movement coordination

## Abstract

In many real-word scenarios, humans and robots are required to coordinate their movements in joint tasks to fulfil a common goal. While several examples regarding dyadic human robot interaction exist in the current literature, multi-agent scenarios in which one or more artificial agents need to interact with many humans are still seldom investigated. In this paper we address the problem of synthesizing an autonomous artificial agent to perform a paradigmatic oscillatory joint task in human ensembles while exhibiting some desired human kinematic features. We propose an architecture based on deep reinforcement learning which is flexible enough to make the artificial agent interact with human groups of different sizes. As a paradigmatic coordination task we consider a multi-agent version of the mirror game, an oscillatory motor task largely used in the literature to study human motor coordination.

## 1 Introduction

The number of scenarios involving humans performing joint tasks with artificial agents is expected to grow rapidly in the near future. Examples, to name just a few, include industrial applications (Hentout et al., 2019), home automation (Miro et al., 2008), assistive and rehabilitation robotics (Freeman et al., 2012), search and rescue tasks (Liu and Nejat, 2013).

While different studies exist in the current literature involving dyadic coordination tasks between one human and one robot or avatar (Lamb et al., 2017; Peternel et al., 2017; Zhai et al., 2017), the problem of developing control-based cognitive architectures to drive autonomous artificial agents to interact with a human team remains much less investigated.

Here, we consider as a paradigmatic example of joint motor task between an avatar and a group of humans a multi-agent version of the *mirror game*. Firstly proposed in the seminal paper by Noy et al. (2011), the mirror game in its original formulation involves two people coordinating the motion of their arm or finger so as to produce synchronous patterns. This task has been largely used in the literature on interpersonal motor coordination and used to develop novel biomarkers for social disorders such as schizophrenia (Slowinski et al., 2014; Zhai et al., 2016; Zhai et al., 2017) but mostly in a dyadic coordination setting. As suggested in Wiltermuth and Heath (2009), indeed, coordination tasks such as the mirror game can be used to help patients affected by mental disorders (e.g., schizophrenia, autism) to improve their social skills. Furthermore, in Slowinski et al. (2014), it was shown that the mirror game can be used to extract the so called Individual Motor Signature (IMS), a time-invariant and unique kinematic signature identifying the motion of each individual.

Following our recent work, e.g., (Lombardi et al., 2018; Lombardi et al., 2019; Lombardi et al., 2021), we consider a multiplayer version of the mirror game where several players are asked to oscillate their end-effector (e.g., a finger if humans) along one direction (e.g., back and forth or sidewise) so as to synchronise their motion while being visually paired with each other (Lombardi et al., 2019; Alderisio et al., 2017a; Alderisio et al., 2017b). We noted that this multiagent version of the game is a suitable task to explore if and how coordination emerges and how it is affected by the configuration of the group and its spatial arrangement [see Alderisio et al. (2017a), Alderisio et al. (2017b) for further details].

A crucial problem when introducing an artificial avatar, or robot, in the group playing the game [as for instance done in Zhai et al. (2016), Zhai et al. (2017)] is to design a control architecture to make the avatar observe the motion of the other group members and coordinate its motion with them in a natural “human-like” way (Lombardi et al., 2019; Lombardi et al., 2021). In this paper we overcome some of the existing limitations on scalability and flexibility of previous proposed designs (Lombardi et al., 2019) by developing an alternative strategy based on deep reinforcement learning. Specifically, our control framework allows the cyberplayer (CP) to perform the task with the others while, at the same time, exhibiting human-like kinematic features. In so doing, our learning algorithm makes the CP emulate the kinematic features in terms of velocity distribution which are typical of the motion of a target human agent while solving the synchronisation problem with the rest of the group. Using observational learning, the CP observes how a target human player performs the group coordination task, extracting some characteristic features of the observed motion and building an internal description model to be used to generate the kinematics of its own motion when replacing the target human player in the group. Effectively, our learning approach is able to make the CP generate new motion at unison with the rest of the group while playing the game with the same kinematic features as those of the target human player it has been programmed to mimic. For the multi-agent case investigated in this paper, we synthesise and validate the control architecture over simulated human models endowed with human features gathered from ad-hoc experimental data.

We wish to emphasise that the novel algorithm we developed to solve this problem can be particularly relevant in those applications, such as health care, where having autonomous artificial agents able to perform coordination tasks with humans can be useful. For example, to enhance the development of exergames involving a mix of human and artificial players coordinating their motion (Freeman et al., 2012; Pirovano et al., 2016).

A preliminary approach to solve the problem was presented in our previous work (Lombardi et al., 2019). In Lombardi et al. (2019), we adopted a different learning approach where the learning agent plays against an “average” player in what boils down to a dyadic interaction between the agent and an average of his neighbours. Therefore, Lombardi et al. (2019) can be seen as an intermediate step between the dyadic case proposed in our earlier works and the multi-agent case investigated in the current manuscript. However, the main drawback of this approach is the assumption that the other players in the group adjust their motion on a real time average of the positions of their neighbours. This is clearly not the case with human players who tend to adjust their motion reciprocally in a number of different ways. To overcome this limitation, the algorithm we present in this paper extracts the main features of the players motion directly from the data. Moreover, to make the approach scalable, we present a training strategy which is independent from the number of players the CP is connected to while playing.

## 2 Previous Work

Using the deep Q-network (DQN) learning algorithm (Mnih et al., 2015), the cyberplayer in Lombardi et al. (2019) was synthesised as an artificial agent able to train itself by observing a specific target player (TP) in order to extract his/her kinematic motor characteristics from the data.

The Deep Q-network strategy exploits an artificial neural network (ANN) to approximate the optimal action-value function Q* characterising the reinforcement learning approach. Contrarily to traditional supervised learning, in the DQN approach the loss function used to train the ANN is iteratively updated through the network’s weights (Russell and Norvig, 2003; Mnih et al., 2015; Sutton and Barto, 2018).

In our setting, the DQN architecture was designed as follows:

• the *state space* is chosen as x≔[x,x,y¯,y¯˙], where $\left[x,x\right]$ are the position and velocity of the CP, while [y¯,y¯˙] the mean position and mean velocity of the neighbours of the target player in the group;
• the *action space* is the set of acceleration values discretised in the range $\left[-u¯,u¯\right]$ with u¯ being the maximum possible acceleration;
• the *reward function* was selected as:

$$\rho ≔-a{\left(x-y_{TP}\right)}^{2}-b{\left(x-y_{TP}\right)}^{2}-\eta u^{2},$$ (1)

where $\left[y_{TP},y_{TP}\right]$ are position and velocity of the target player, *u* is the control action, the constant weights *a* = 1 and *b* = 0.1 are used to tune the position error and the velocity error respectively, while the constant weight *η* = 10^–4^ is used to tune the control effort;

• the *policy* *π* is an *ϵ*-greedy policy as in Sutton and Barto (2018);
• the neural network considered to approximate the action-value function *Q* was designed as a fully connected [64, 32] feed-forward network with 4-nodes input layer (one node for each state variable) and 9-nodes output layer (one node for each action value).

The main drawback in the solution proposed in Lombardi et al. (2019) is the explicit use of the mean of the position and velocity of the neighbours as variables in the state of the CP. Considering such a feature it is implicitly assumed that a human player first estimates the mean of his/her neighbours and then tries to minimise the error between himself/herself and such estimated mean.

In the next section, to overcome this issue, we will remove such an assumption in the design and implementation of the CP. Specifically, the whole state of the neighbours of the CP will be considered and used as input to the neural network, leaving to the learning algorithm the task of extracting the main kinematic features of the target player the CP is asked to emulate.

## 3 Cyber Player Synthesis

### 3.1 Architecture

As already recalled in Section 1, the aim of this paper is to design a CP able to learn and exhibit the same motor kinematic features of a target agent when playing the mirror game task with a group of other agents. The group of interacting agents is implemented through the formalism of complex networks, where each agent is represented as a node while the visual coupling with the others as edges in the graph.

The problem is formalised by considering a set *X* of all possible states in which the environment can be (state-space), a set *U* of all possible actions that the agent can take (action-space), an auxiliary function *Q* that estimates the value of taking a specific action from a specific state in terms of expected returns defined by a reward function. Specifically the *action space* *U* and the *policy* *π* are defined as in Lombardi et al. (2019) and reported in Section 2, while the reward function and the state space are detailed as follows:

• the weights of the *reward function* in Eq. 1 are selected empirically to maximize the performance of the CP and they are *a* = 0.7 and *b* = 0.3;
• the *state space* is the vector x≔[y,y˙,Δy,Δy˙], where the subvector $\left[y,y˙]≔[y_{i},{y˙}_{i}\right]$ with *i* = 1, … , *N* is the position and the velocity of the neighbours of the CP, while the subvector $[\Delta y,\Delta y˙]≔\left[\left(x-y_{i}\right)],[\left(x˙-{y˙}_{i}\right)\right]$, with again *i* = 1, … , *N*, is the error in position and in velocity between the CP and each neighbour *i*. *N* is the number of the neighbours of the CP, i.e., the number of group members the CP is directly connected with.

A specific challenge of the proposed architecture is that the state space of the CP changes depending on the number of its neighbours and hence, on the specific network topology connecting the players in the group. In order to have a cyberplayer able to play the mirror game in any group configuration (i.e., with any number of neighbours, say *M*, up to a maximum of, say, *N*), we consider a fixed size state space vector capable of supporting *N* neighbours. We denote with M the set of the effective neighbours of the CP, and with *M* ≤ *N* its cardinality. Notice that such a hypothesis is not restrictive, as *N* can be chosen arbitrarily.

Specifically, considering for each *i* = 1, … , *N*:

• if player i∈M, the subvector $\left[y_{i},{y˙}_{i},\left(x-y_{i}\right),\left(x˙-{y˙}_{i}\right)\right]$ will be included in the state vector of the CP;
• if player i∉M, the subvector [x,x,0,0] will be included in the state vector of the CP. We term such player *i* as a “ghost” neighbour. Notice that, setting the subvector corresponding to the ghost neighbour with the same position and velocity vector of the CP, means that such subvector will not contribute to the computation of the reward function and therefore will not influence any decision made by the CP.

**FIGURE 1:**
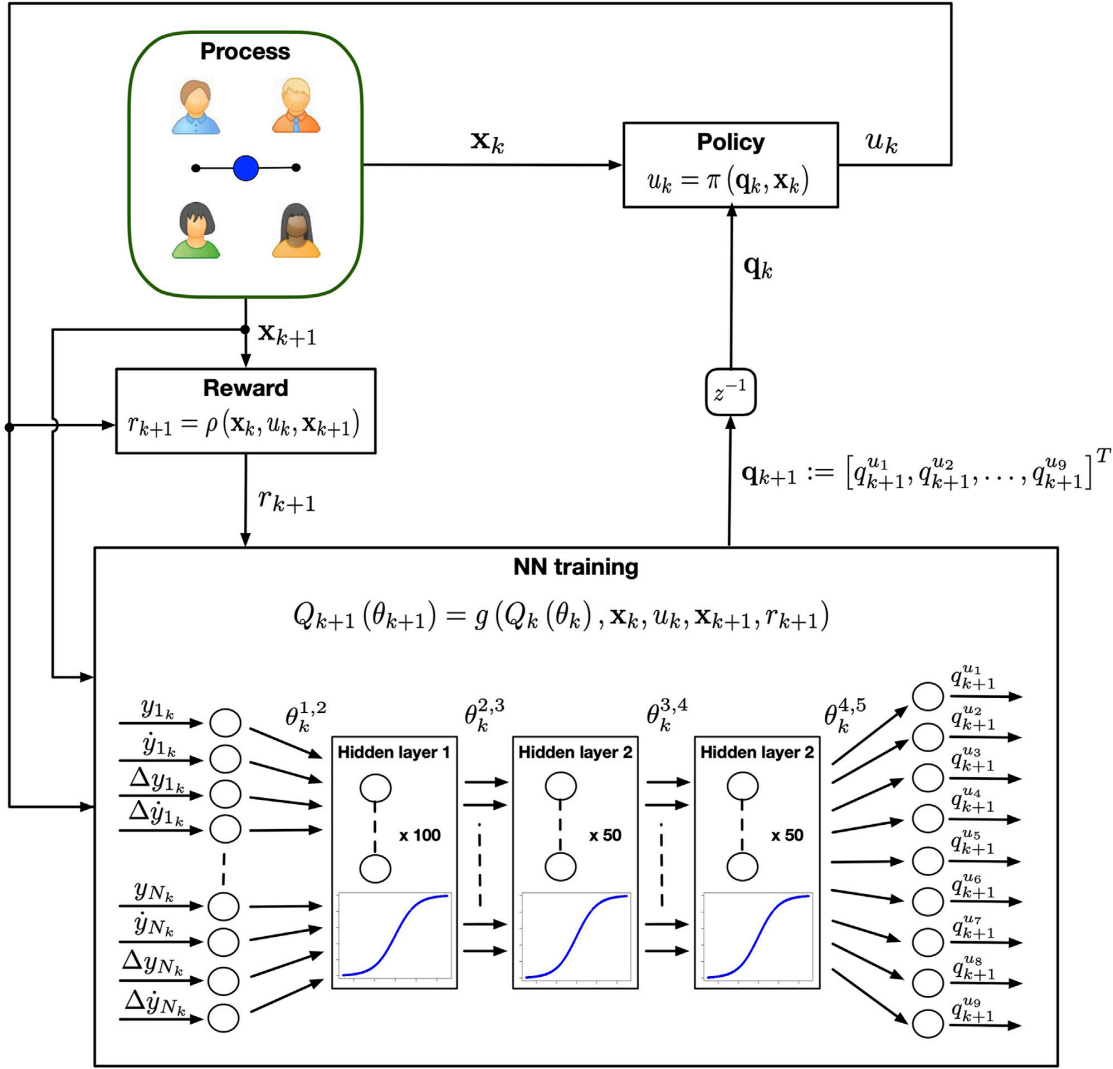
Control architecture of the cyber player playing the mirror game in a group. At each iteration *k*, the DQN controller observes the state of the game and chooses the control input *u* according to the estimated neural network. The process evolves in a new state and the CP receives a reward *r*. The set of reward, previous and current state are then used to update the weights of the neural network.

The ANN considered to approximate the *Q* function is designed as a feed forward network with (Figure 1):

• an *input layer* with *N* different nodes representing the maximum number of players connected to the CP and hence the dimension of the stack state vector;
• *three hidden layers*, made of 100, 50 and 50 nodes respectively, each implementing a sigmoidal activation function. The number of layers and that of their nodes were found heuristically by trial-and-error to maximise the performance and convergence time of the learning algorithm;
• an *output layer* with nine different nodes, one for each action variable in the action space. The neural network returns an action-value *q* ^*u*^ for each action available in the set *U*. Then, the action corresponding to the maximum *q*-value is chosen as control input.

### 3.2 Implementation

The Deep Q-network algorithm is known to be unstable or even to diverge when a nonlinear function approximator (ANN) is used to estimate the *Q*-function (Mnih et al., 2015; Sutton and Barto, 2018). Such instability is caused by the presence of correlation in the observation sequence and between the estimated network *Q* and the optimal target network Q*, resulting in the loss of the Markov property. To overcome this issue, the correlation in the observation sequence is removed by introducing an *experience replay mechanism*, where the observed states used to train the ANN are not taken sequentially but are sampled randomly in batch from a circular buffer (Mnih et al., 2015). Also, the correlation between the current estimate of the function *Q* and the target optimal network Q*, used in the loss function, is reduced updating the latter at a slower rate instead of at each iteration.

In the DQN the loss function is iteratively changed because the predicted output itself depends on the network parameters *θ*_*k*_ at every instant *k*. Namely, the loss function is chosen as:

$$L_{k}\left(\theta_{k}\right)=E\left[{\left(r_{k}+\gamma \;\underset{u_{k+1}}{\max}\;Q\left(x_{k+1},u_{k+1},\theta_{k-1}\right)-Q\left(x_{k},u_{k},\theta_{k}\right)\right)}^{2}\right],$$ (2)

which represents the mean squared error between the current estimated *Q* function and the approximate optimal action-value function.

After having initialised the ANN with random values and instantiated an empty buffer for the experience replay mechanism, the training of the CP is performed iteratively until convergence is achieved according to the following “termination criterion”: $\left\|RMS_{TP,y_{i}}-RMS_{CP,y_{i}}\right\|\leq \epsilon \;\forall i\in M$, where $RMS_{TP,y_{i}}$ and $RMS_{CP,y_{i}}$ are the root mean square error between the position of the neighbour *i* and, correspondingly, the position of the CP and the target player, while *ϵ* is a non-negative parameter.

Our algorithm reports a time complexity of *O*(*N*) where *N* is the number of nodes (players) in the network. Specifically, let the complexity of the first layer of a feed forward NN be *O*(*P* ⋅ *M*) where *P* is the cardinality of the input layer (number of the neighbours) and *M* is the number of the hidden nodes of the first layer. Since we consider *M* constant, we have *O*(*P* ⋅ *M*) = *O*(*P*). Also, considering that the ghost neighbours do not play a role in the NN, the major contribution to the complexity comes from the number of the effective neighbours (independently from the implemented topology). Hence, in the worst case the number of neighbours *P* = *N* − 1 and so the resulting complexity is *O*(*P*) = *O*(*N* − 1) = *O*(*N*).

## 4 Training Setup and Validation

### 4.1 Training

As the learning process typically requires a very large dataset, real data acquired during live sessions of the mirror game between human players might be difficult to collect.

To overcome this problem, we use a practical way to train the CP, proposed in Lombardi et al. (2019), Lombardi et al. (2021). In this setup, enough synthetic data are generated by making several “virtual trainers” (VT) perform group sessions of the mirror game against each other. Each VT is driven by a model-based controlled architecture embedding in its core a stochastic model capturing human-like kinematic characteristics [see Lombardi et al. (2018) for more details]. Specifically the motion of the VT is generated by a controlled nonlinear HKB oscillator (Haken et al., 1985):

$$x¨+\left(\alpha x^{2}+\beta x^{2}-\gamma \right)x+\omega^{2}x=u,$$ (3)

where x,x˙ and x¨ are position, velocity and acceleration of the VT, respectively, *α*, *β*, *γ* are positive empirically tuned damping parameters while *ω* is the natural oscillation frequency. The control input *u* is chosen as solution of an optimal control problem having the following cost function (Zhai et al., 2016):

$$\begin{array}{l} \underset{u}{\min}J\left(t_{k}\right)=\frac{\theta_{p}}{2}{\left(x\left(t_{k+1}\right)-{r¯}_{p}\left(t_{k+1}\right)\right)}^{2}+\frac{\theta_{\sigma }}{2}\int_{t_{k}}^{t_{k+1}}{\left(x˙\left(\tau \right)-{r˙}_{\sigma }\left(\tau \right)\right)}^{2}\;d\tau +\\ \frac{\theta_{v}}{2}\int_{t_{k}}^{t_{k+1}}{\left(x˙\left(\tau \right)-{r¯˙}_{p}\left(\tau \right)\right)}^{2}\;d\tau +\frac{\eta }{2}\int_{t_{k}}^{t_{k+1}}u{\left(\tau \right)}^{2}\;d\tau , \end{array}$$ (4)

where ${r¯}_{p},{r¯˙}_{p}$ are the mean values of the position and the velocity of the VT’s neighbours, i.e., the agents it is connected with, *η* tunes the control effort, [*t*_*k*_, *t*_*k*+1_] represents the optimisation interval, while *r*_*σ*_ is the reference signal coming from a stochastic model of the Markov chain (MC) aiming at modelling the human individual motor signature and derived from data gathered from ad-hoc experimental sessions [as done in Lombardi et al. (2018), Lombardi et al. (2021)]. Finally, *θ*_*p*_, *θ*_*s*_, *θ*_*v*_ are positive control parameters satisfying the constraint *θ*_*p*_ + *θ*_*s*_ + *θ*_*v*_ = 1. By tuning appropriately these parameters, it is possible to change the VT configuration making it act as a leader, follower or joint improviser in the mirror game [more details are in Zhai et al. (2016), Zhai et al. (2017)]. It has been proved that the MC-based control architecture can be carefully tuned such that the VT generates trajectories with the same kinematic characteristic of the human player on which the Markov chain has been trained (Zhai et al., 2017; Lombardi et al., 2021). The main advantage of such a training approach is that with few virtual trainers it is possible to synthesise a cyber player general enough to play the mirror game with any player while exhibiting the desired human motor signature. The use of virtual trainers is a simple method to generate as much synthetic data as needed by the learning algorithm. Note that the offline tuning parameters is needed only for the virtual trainers used during the training.

To train the CP to emulate a target VT while coordinating its movements in the group, we built a group of four different VTs performing trials of the mirror game while interconnected through a random graph. A new random graph is generated at each training trial. Each VT was synthesised and ad-hoc parameterised in order to emulate the behaviour of the human player whose trials were used to train the Markov chain embedded in its architecture. In particular, we experimentally built six different MCs (one for each VT) and parameterised each VT_*i*_ (*ω*, *θ*_*p*_, *θ*_*s*_) as follows: VT_1_: (*ω* = 0.75, *θ*_*p*_ = 0.8, *θ*_*s*_ = 0.15); VT_2_: (*ω* = 0.4, *θ*_*p*_ = 0.8, *θ*_*s*_ = 0.15); VT_3_: (*ω* = 0.5, *θ*_*p*_ = 0.8, *θ*_*s*_ = 0.15); VT_4_: (*ω* = 0.75, *θ*_*p*_ = 0.8, *θ*_*s*_ = 0.15); VT_5_: (*ω* = 1, *θ*_*p*_ = 0.75, *θ*_*s*_ = 0.2); VT_6_: (*ω* = 0.8, *θ*_*p*_ = 0.85, *θ*_*s*_ = 0.1); VT_7_: (*ω* = 0.5, *θ*_*p*_ = 0.75, *θ*_*s*_ = 0.2). The parameters (*α* = 1, *β* = 2, *γ* = −1, *θ*_*v*_ = 0.05, *η* = 10^–^^4^) were set equal to all VTs.

In the deep learning algorithm the CP was trained to emulate VT_4_ (any other VT can be used). In particular the group with VT_1_, VT_2_, VT_3_ and VT_4_ was used during the training, whereas the group VT_4_, VT_5_, VT_6_ and VT_7_ was used for the validation.

The experience replay was implemented with a buffer of 200.000 elements, batches of 32 sampled states were used to train the feed forward neural network at each iteration. A target network updated every 150 time steps was considered in the *Q*-function, with a discount factor *γ* = 0.95 and a learning rate of 0.1.

**FIGURE 2:**
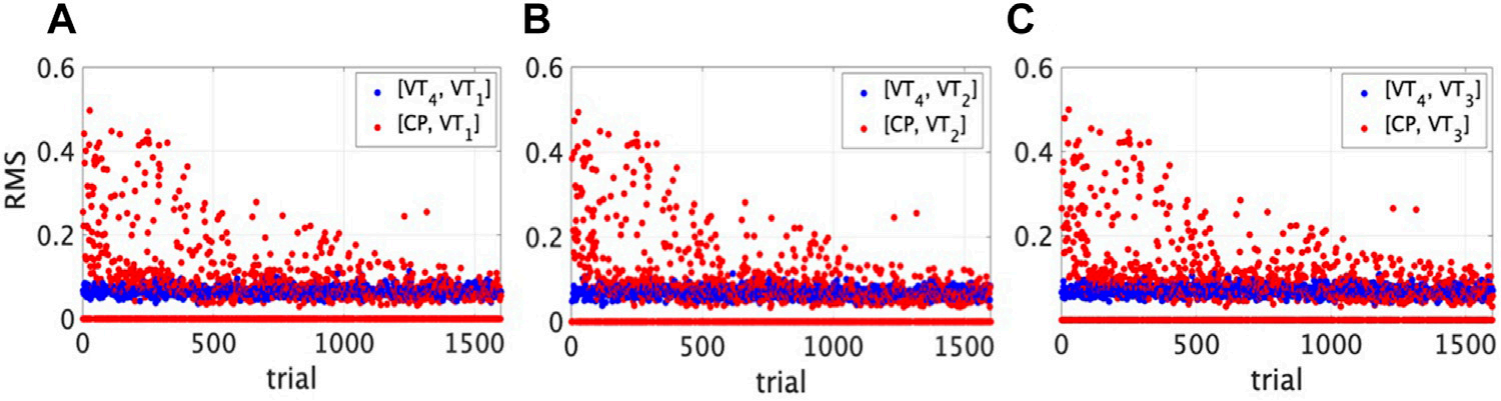
Training curve showing the convergence of the algorithm. The root mean square error in position (*y*-axis) is reported for each trial (*x*-axis) both between the target VT_4_ (in blue) and **(A)** VT_1_, **(B)** VT_2_, **(C)** VT_3_, and between the CP (in red) and the same players.

In Figure 2 the training curve is reported showing for each trial the RMS error of the position between the VT and each neighbour (in blue), and between the CP and the same neighbour (in red).

### 4.2 Validation

**FIGURE 3:**
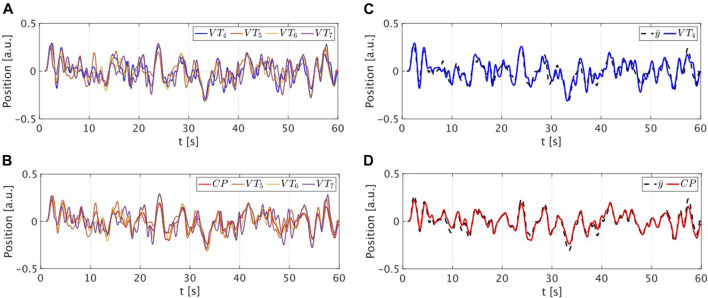
Sample trial of a group session. **(A,B)** Position time series of the target VT_4_ and of the CP while playing with the other virtual trainers. Different colours are used for different players. **(C,D)** Position time series of the target VT_4_ (in blue) and of the CP (in red) reported together with the mean position of the neighbours (in dashed black).

The validation was performed comparing the performance of the CP with that of the target VT. Specifically VT_4_ and CP performed 60 trials of 60 s of the mirror game connected with VT_5_, VT_6_ and VT_7_ in a random graph. A new random graph was generated at each trial. A sample session trial is depicted in Figure 3. The CP successfully tracks the mean position of the group meaning that it has correctly learned the same strategy implemented by the virtual trainers. Notice that such a strategy was not encoded in the CP, which learned it by only observing the target VT and its neighbours.

Quantitatively, the performance was evaluated in terms of:

1) *relative phase error* defined as $\Delta \Phi =\Phi_{y¯}-\Phi_{CP/VT_{4}}$, where $\Phi_{CP/VT_{4}}$ is the phase of the CP and VT_4_ respectively while $\Phi_{y¯}$ is the average of that of the neighbours. The phase was estimated taking the Hilbert transform of the corresponding position signal (Kralemann et al., 2008);
2) *RMS error* between the position time series of the CP (or VT_4_) and the mean position time series of its neighbours;
3) *time lag* which describes the amount of time shift that achieves the maximum cross-covariance between the position time series of the CP (VT_4_) and the average of that of the neighbours. This can be interpreted as the average reaction time of the players (Orfanidis, 1988). Since the maximum cross-covariance achieved by the CP and VT_4_ can be highly different from each other while keeping same time lag values, we reported also the maximum cross-covariance *K* in position as metric of interest;
4) *group synchronisation index* introduced in Richardson et al. (2012); Alderisio et al. (2017a) and defined as:

$$\rho_{g}\left(t\right)≔\frac{1}{P}\left|\overset{P}{\underset{k=1}{\sum }}e^{j\left(\phi_{k}\left(t\right)-{\phi ¯}_{k}\right)}\right|\;\in \left[0,1\right],$$ (5)

where $\phi_{k}\left(t\right)≔\theta_{k}\left(t\right)-q\left(t\right)$ is the relative phase between the *k*th player and the group phase at time *t*, ${\bar{\phi }}_{k}$ is *ϕ*_*k*_(*t*) averaged over time, and *P* is the number of the players. The closer the synchronisation index is to 1, the higher is the level of synchronisation in the group.

The number of trials chosen for the validation was the result of the statistical power analysis carried out taking as metric the group synchronisation index and a reference power of 0.9. Mean and standard deviation are reported over the total number of trials for each metric both for VT_4_ and for CP. Before running any statistical test, we removed the outliers classifying them as the data points that were 2.5 times the standard deviation away from the mean. Since the data were not normally distributed, we performed the Wilcoxon *t*-test as a non-parametric test reporting the following results:

• Relative phase error ΔΦ. CP: − 5.127*e* ^−4^ ± 0.032; VT_4_: −2.506*e* ^−4^ ± 0.023 (W (54) = 732, p = 0.753, effect-size = −0.049).
• RMS position error. CP: 0.062 ± 0.018; VT_4_: 0.054 ± 0.006 (W (54) = 606, p = 0.171, effect-size = −0.213).
• Time lag. CP: − 0.021 ± 0.031; VT_4_: −0.034 ± 0.051 (W (54) = 469.5, p = 0.096, effect-size = −0.264).
• Maximum cross-covariance *K*. CP: 0.881 ± 0.064; VT_4_: 0.887 ± 0.024 (W (54) = 801, p = 0.798, effect-size = −0.040).
• Group synchronisation index *ρ*_*g*_. CP: 0.821 ± 0.086; VT_4_: 0.804 ± 0.046 (W (54) = 593, p = 0.139, effect-size = −0.230).

**FIGURE 4:**
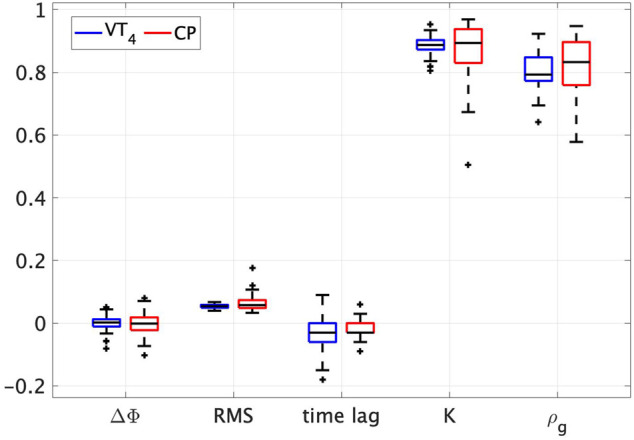
Boxplots of the metrics used to validate the CP. Two boxplots are shown for each metric used to validate the learning algorithm (relative phase, RMS position error, time lag, max cross-covariance and synchronisation index). In blue those of the VT_4_, in red those of the CP.

A *p*-value > 0.05 was computed for all the metrics of interest showing that no significant difference exists between the CP and the player it is emulating (boxplots are depicted in Figure 4). Codes and data can be found at https://github.com/diBernardoGroup/CyberPlayer_DQN/.

## 5 Conclusion

In this work, we addressed the problem of synthesising an autonomous artificial agent (called cyber player) able to coordinate its movement and perform a joint motor task in a group scenario. In particular, a multiplayer version of the mirror game was used as oscillatory joint task. To achieve our goal we used a DQN approach to train the CP taking as input the state (position and velocity) of its neighbours in the network. Contrarily to what we have previously done in Lombardi et al. (2019), where the mean position of the neighbours was extracted a priori and used as input to the neural network, in the proposed architecture we overcame this limitation by letting the learning algorithm extract directly from the data the strategy implemented by the players. To avoid that such an approach could lead to an undesired dependency of the CP on a specific network topology and making the algorithm not general for any network, we approached the problem by sizing the CP neighbours to a maximum number. Such value is a control parameter that can be selected according to the connectivity we aim at designing for the CP. In the case the CP has an effective lower number of connected agents, we increase the number of neighbours artificially by introducing “ghost neighbours” that do not alter the learning and decision process but allow the algorithm to cope with randomly selected network structures (and hence a random number of CP’s neighbours up to *N*).

The effectiveness of the algorithm was shown numerically by comparing its performance with that of a target VT while playing in a group of four human emulating agents over different group configurations. Furthermore, statistical analysis proved that no statistical difference exists between the CP and the target VT therefore showing that the CP is effectively able to perform motor interactions in a group with the same motor features exhibited by the target agent. Ongoing work is being carried out to validate the CP when interacting with a real group of people in an experimental setting, as already done for the dyadic interaction in Lombardi et al. (2021).

## Data Availability

The dataset and the code used in this study are publicly available at https://github.com/diBernardoGroup/CyberPlayer_DQN/.
